# Analysis of MWCNT/epoxy composites at microwave frequency: reproducibility investigation

**DOI:** 10.1186/1556-276X-9-168

**Published:** 2014-04-05

**Authors:** Mauro Giorcelli, Patrizia Savi, Mario Miscuglio, Muna Hajj Yahya, Alberto Tagliaferro

**Affiliations:** 1 Department of Applied Science and Technology (DISAT), Politecnico di Torino, Corso Duca degli Abruzzi, 24, Torino 10129, Italy; 2 Department of Electronic and Telecommunication (DET), Politecnico di Torino, Corso Duca degli Abruzzi, 24, Torino 10129, Italy

**Keywords:** Carbon nanotubes, Epoxy resin, Permittivity measurements

## Abstract

A wide-band microwave characterization of nanocomposites based on commercial multiwalled carbon nanotubes (MWCNTs) and epoxy resin is presented. The sample preparation method is discussed in detail. Field emission scanning electron microscopy is used for morphological sample analysis of nanocomposites and MWCNTs. The complex permittivity is measured in a wide frequency band (3 to 18 GHz) using a commercial dielectric probe (Agilent 85070D) and a network analyzer (E8361A). A statistical analysis based on one-way analysis of variance (ANOVA) technique is performed. The aim of this statistical analysis is to investigate the influence of concentration of nanoparticles inside the polymer matrix on the complex permittivity. This can be significantly different in nanocomposites even if the samples have similar electrical properties.

## Background

Nanocomposites (NCs) are the new frontier of materials in civil and military applications. In particular, polymer NCs are a hot spot in several research fields. As a general rule, NCs are prepared by dispersing a nanometer-sized filler into a polymer matrix creating a network able to improve the properties of a host polymer.

Carbon nanotubes (CNTs) and, in particular, multiwalled CNTs (MWCNTs) have been used intensively as a filler in a variety of polymers [1,2]. Their outstanding mechanical, electrical, and thermal properties allow then to enhance the properties of the material in which they are used as a filler for matrix reinforcement [3]. Also, this increase in performance takes place even at low percentages of MWCNTs. A critical point is the MWCNT dispersion as reported by Bauhofer [4] because with an accurate dispersion, it is possible to lower the MWCNT amount required to improve host material performances. Recently, MWCNT composites have been proposed as microwave absorbers [5,6] and for shielding applications [7-10]. For these applications, the ability to tailor the values of complex permittivity with characteristics of the matrix and MWCNT concentration is critical.

In this work, NCs based on MWCNTs and epoxy resin were prepared using an *in situ* polymerization process. Special care was paid to avoid any imperfection in dispersion or defects.

The complex permittivity of epoxy resin and NC with 1 and 3 wt.% MWCNTs was measured in the frequency range 3 to 18 GHz using a commercial dielectric probe (Agilent 85070D; Agilent Technologies, Sta. Clara, CA, USA) and a network analyzer (E8361A; Agilent Technologies). The sample's reproducibility was tested applying a statistical analysis based on a one-way analysis of variance (ANOVA) technique.

## Methods

In the NC fabrication process, one kind of MWCNT (NTX-3; Nanothinx, Rio Patras, Greece) was used as a filler at 1 and 3 wt.% concentrations. The nominal MWCNT characteristics were diameter 25 to 45 nm, length >10 μm, purity >98%. The nominal aspect ratio thus varies from 250 to 400 where an average of 325 is assumed in the following process.

Epilox, a commercial thermosetting resin produced by Leuna-Harze (Leuna, Germany) was used as polymer matrix. It is a bi-component system formed by a resin and a hardener. Resin (T-19-36/700) is a modified commercial matter, colorless, and low-viscosity (650 to 750 mPa s at 25°C) epoxy resin with reduced crystallization tendency with a density of 1.14 g cm^-3^. The chemical composition of Epilox resin T19-36/700 is mainly bisphenol A (30 to 60 wt.%), with an addition of crystalline silica (quartz) (1 to 10 wt.%), glycidyl ether (1 to 10 wt.%), and inner fillers (10 to 60 wt.%). The hardener (H10-31) is a liquid, colorless, and low-viscosity (400 to 600 mPa s at 25°C) modified cycloaliphatic polyamine epoxy adduct with a density of 1 g cm^-3^.

**Figure 1:**
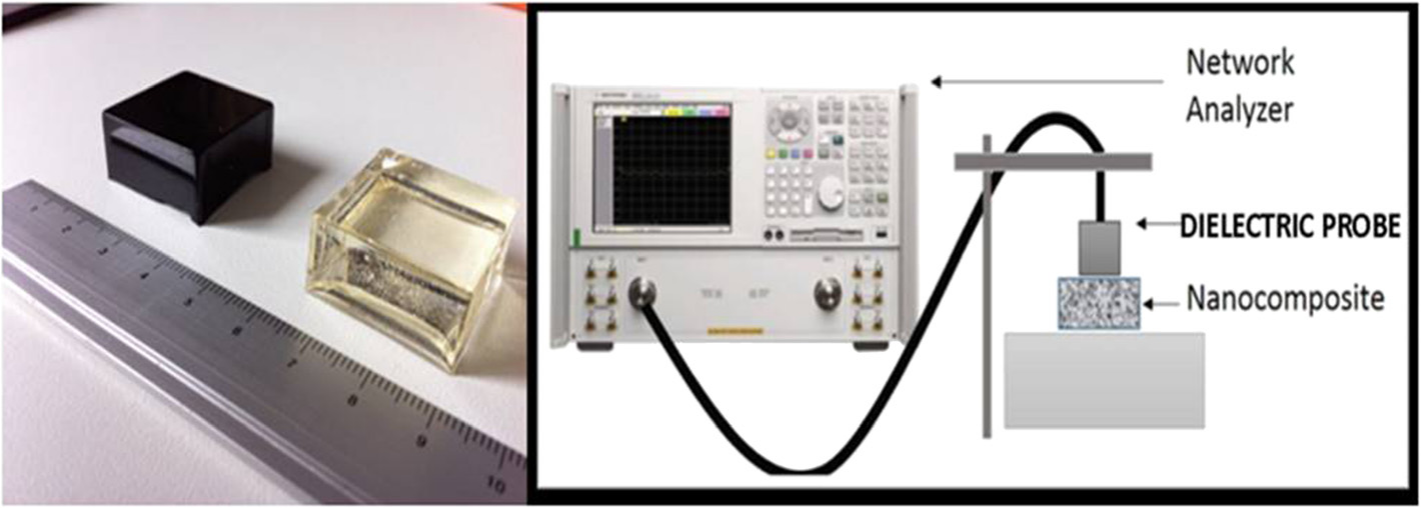
**Image of NC and sketch of the setup.** Left panel: image of NC (pristine epoxy resin reinforcement) (black) and polymer (pristine epoxy resin) (transparent). Right panel: sketch of the measurement setup.

In the first step, after the weighing of these two compounds, the resin was mixed with the MWCNTs using a high-shear T-25 ULTRA-TURRAX® (IKA, Rawang, Selangor, Malaysia) mixer for 2 min. This mixer guarantees a high and homogeneous mechanical dispersion of the carbon filler inside the resin. Material dispersion is a crucial point in order to obtain a uniform performance of the final product. In the second step, the hardener was added to resin/MWCNT composite and mechanically mixed at 1,200 rpm for approximately 5 min. The final composites were poured into moulds once good dispersion was achieved. The shape and the thickness of the samples (see Figure 1, left panel) were chosen in order to fulfill the requirements of the setup of the complex permittivity measurements. The moulds filled with the composite were placed in a vacuum chamber to remove all air bubbles in the samples due to mixing. The samples were then cured in the oven at 74°C for 4 h in order to speed up the polymerization, as prescribed by the polymer datasheet. In Figure 1 (left panel), real-scale images of 1 wt.% MWCNTs/epoxy (black specimen) and pristine epoxy (transparent specimen) are shown.

As the dispersion of MWCNTs inside the resin is a crucial point, it was checked using field emission scanning electron microscopy (FESEM; Zeiss Supra 40; Carl Zeiss AG, Oberkochen, Germany) by analyzing the exposed surfaces of the crio-fractured samples. Breaking the specimen into two pieces after flash-freezing in liquid nitrogen guaranteed that the internal structure was not affected by the fracture, avoiding internal resin elongation with subsequent MWCNT reorientation.

To obtain high values of the real part of permittivity, the volume fraction should be above the percolation threshold [10]. For long fibers with large aspect ratio (AR), the volume fraction value at the percolation threshold can be approximately evaluated as 1/AR [4,9,11]. Consequently, for the MWCNTs used in this work, we can estimate a value around 0.3 vol.%. The volume fractions *φ* were obtained from the weight fractions of MWCNTs using the densities of MWCNTs (*ρ*_MWCNTs_ = 2.05 g cm^-3^), the polymer matrix (*ρ*_poly_ = 1.3 g cm^-3^) and their weight ratio *x*, as reported in [12]:

(1) $$\varphi =\frac{\rho_{\mathrm{poly}}}{\rho_{\mathrm{CNTs}}+\rho_{\mathrm{poly}}x}$$

In our investigation, 1 and 3 wt.% correspond to 0.64 and to 1.92 vol.%, respectively. In both cases, the volume fraction was above the percolation threshold.

Further, considering time-harmonic fields, constitutive elements are a complex numbers and a complex permittivity which can be defined as ϵ = ϵ - *jγ*/*ω* = ϵ′ - *j*ϵ″, with *γ* being the conductivity and *ω* the angular frequency [13]. In fact, if an electromagnetic field propagates in a loss dielectric material, two kinds of electrical currents arise: displacement and conduction currents. The real part of permittivity describes the polarization effect due to the interaction with bound charges (i.e., the displacement current), and the imaginary part describes the effects due to free electron's (conduction current) increase to power loss.

The complex permittivity of pure epoxy resin and composites with 1 and 3 wt.% MWCNTs was measured in the frequency range of 3 to 18 GHz. The samples were measured using a commercial dielectric probe (Agilent 85070D) and a network analyzer (E8361A). The measurement setup is shown in Figure 1 (right panel). A standard calibration short/air/water was adopted. This type of measurements was chosen because of its wider-band feasibility (200 MHz to 20 GHz) with respect to waveguide measurements or free-space measurements; moreover, the samples can be of relatively small dimensions. The drawback is that samples should have a very smooth and flat surface in order to avoid the presence of an air gap at the probe face [14,15].

The electrical properties of the polymer were tailored by changing the concentration of MWCNTs. Four different specimens were prepared for each concentration of MWCNTs in order to give statistical significance of the permittivity results. The differences among the two concentrations of MWCNTs (1 and 3 wt.%) and pristine epoxy resin were tested through the one-way ANOVA technique. The one-way ANOVA compares the means between the groups (i.e., the different concentrations) and determines the level of significance of the null hypothesis. This method allows us to determine the impact of the nanoparticles on the electrical properties of the composites. By applying Tukey's multiple comparison tests to the data a level of confidence, *p* value was estimated for each compared pair (*p* > 0.05, *p* ≤ 0.01, *p* ≤ 0.001). The standard deviation of measurements performed on four samples is represented by error bars. The number of samples considered is representative of the statistical calculation, because the conditions of the ANOVA test (independence of the samples, normality of the data points among the population, absence of outliers in the population, and almost equality of population variances) hold. This analysis was performed with Graphpad Prism® (GraphPad Software, Inc., La Jolla, CA, USA).

## Results and discussion

**Figure 2:**
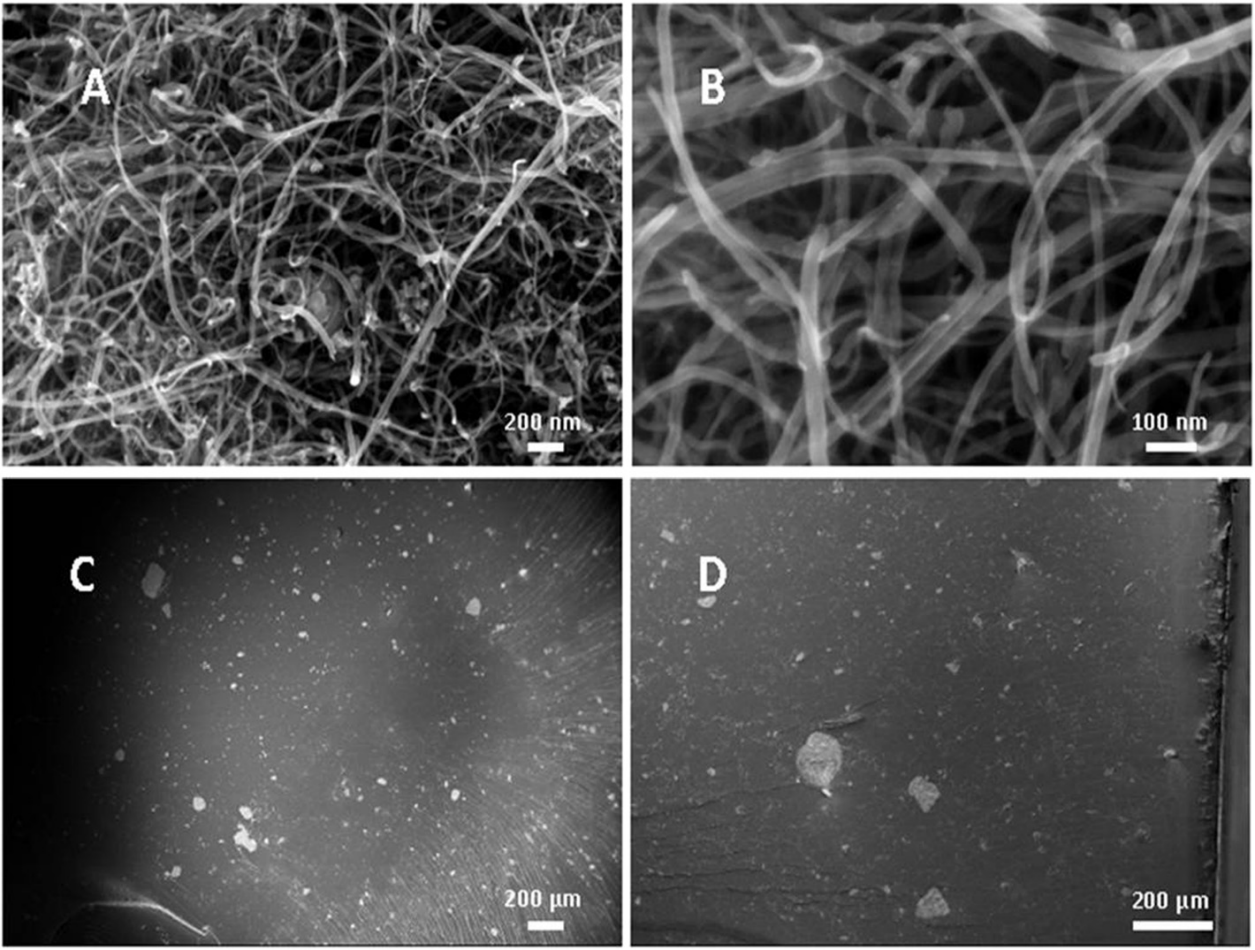
**FESEM images of MWCNTs and crio-fractured area of NC.** FESEM images of used MWCNTs **(A, B)** and crio-fractured area of the NC at 1 wt.% **(C)** and 3 wt.% **(D)** of MWCNTs.

FESEM analysis was performed on MWCNTs and for several crio-fractured surfaces and the results are reported in Figure 2. As shown in Figure 2A, MWCNTs were so entangled and some impurities were present. Long MWCNTs were subjected to bull up, and this increases the difficulty to obtain a uniform dispersion. As shown in Figure 2C,D, several agglomerates less than 100 μm in size were present, and they were uniformly distributed inside the NC.

**Figure 3:**
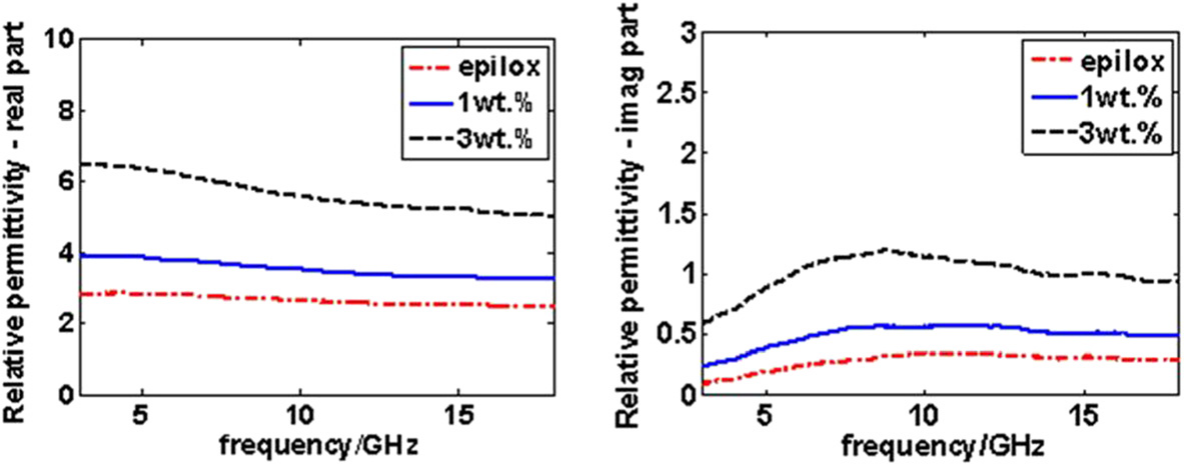
**Relative permittivity of studied NC.** Left, real part. Right, imaginary part.

The real and imaginary parts of the measured complex permittivity for pristine Epilox resin and NC with 1 and 3 wt.% of MWCNTs are reported in Figure 3. As expected, the increasing of filler concentration increases the value of both real and imaginary parts.

**Figure 4:**
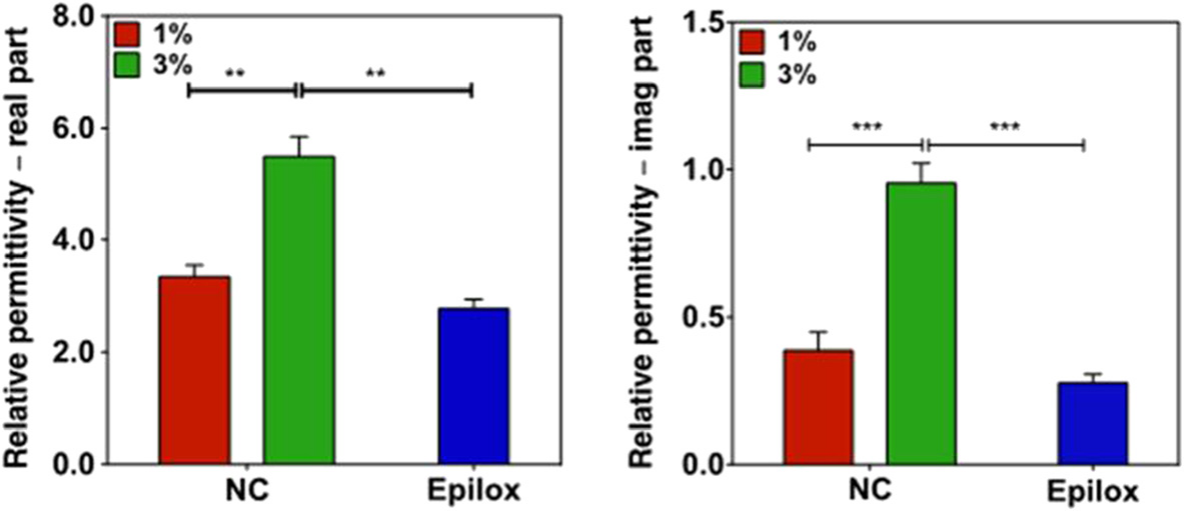
**Statistical analysis.** **p ≤ 0.01; ***p ≤ 0.001. Error bars represent the standard deviation of measurements performed.

Concerning the statistical analysis, the graph in Figure 4 (left) shows that there is no statistically significant difference between the Epilox resin and the NC (1 wt.% MWCNTs) in terms of real part of relative permittivity, (*p* > 0.05). However, the higher MWCNT concentration (3 wt.%) leads to a statistically significant difference in comparison to both the normal Epilox resin (without MWCNTs) and NC (with 1 wt.% MWCNTs) (*p* ≤ 0.01). The bar chart in Figure 4 (right) highlights how the imaginary part of the permittivity increases by increasing the concentration. The difference between the pristine epoxy resin and the NC (1 wt.% MWCNTs) proves that a small concentration of MWCNTs was not sufficient to produce a significant variation in the imaginary part of the permittivity (*p* > 0.05). On the other hand, incorporating more, such as 3 wt.% of MWCNTs, inside the epoxy resin, significantly improves the imaginary part of the permittivity that is strictly related to NC conductivity (*p* ≤ 0.001). Lastly, it was revealed that a concentration of 3 wt.% of MWCNTs is able to significantly increase both the imaginary and the only real parts of the permittivity (*p* ≤ 0.001). Details of the results are shown in Tables 1 and 2, respectively. In the second column, the mean difference of the comparison between the pairs under examination is shown, while in the third column, the 95% confidence interval (CI) of the mean difference is given.

**Table 1:** Multiple comparison summary - relative permittivity - real part

**Tukey’s multiple comparison tests**	**Mean diff**	**95% CI of diff**	**Adjusted *p *** **value**	**Significant?**	**Summary**
1 wt.% vs. 3 wt.%	-2.186	-2.865 to -1.507	0.0031	Yes	**
1 wt.% vs. Epilox	0.5255	-0.09689 to 1.148	0.1233	No	ns
3 wt.% vs. Epilox	2.712	1.870 to 3.553	0.0027	Yes	**

**p ≤ 0.01, ns, not significant; diff, difference.

**Table 2:** Multiple comparison summary - relative permittivity - imaginary part

**Tukey’s multiple comparison tests**	**Mean diff**	**95% CI of diff**	**Adjusted *p *** **value**	**Significant?**	**Summary**
1 wt.% vs. 3 wt.%	-0.5777	-0.6655 to 0.4899	0.0002	Yes	***
1 wt.% vs. Epilox	0.1014	-0.0446 to 0.2474	0.1265	No	ns
3 wt.% vs. Epilox	0.6792	0.5381 to 0.8202	0.0006	Yes	***

***p ≤ 0.001; ns, not significant; diff, difference.

## Conclusions

Nanocomposites based on epoxy resin and MWCNTs in two different concentrations were made. FESEM analysis showed a discrete dispersion of MWCNTs inside material. As it was expected, the complex permittivity measured in the frequency range of 3 to 18 GHz increases with filler concentration. The statistical analysis of variance, using ANOVA technique, showed that there was no difference between pristine epoxy resin and NC with 1 wt.% of MWCNTs. The difference in permittivity, real and imaginary part, is significant only with 3 wt.% of MWCNTs. Future works will be on the application of this analysis to other types of MWCNTs in order to consolidate the present data.

## Abbreviations

CN: Carbon nanotube; MWCNT: Multiwalled carbon nanotube; NC: Nanocomposites.

## Competing interests

The authors declare that they have no competing interests.

## Authors’ contributions

MG and HYM carried on the samples preparations. PS and HYM the permittivity measurements, MM performed the statistical analysis. PS, MM and AT analyzed and interpreted the data. MG, MM and PS wrote the manuscript. All authors were involved in the critical discussions and revision of the manuscript. All authors read and approved the final manuscript.
